# β-catenin and transforming growth factor β have distinct roles regulating fibroblast cell motility and the induction of collagen lattice contraction

**DOI:** 10.1186/1471-2121-10-38

**Published:** 2009-05-11

**Authors:** Raymond Poon, Saeid Amini Nik, Jessica Ahn, Laura Slade, Benjamin A Alman

**Affiliations:** 1 Program in Developmental and Stem Cell Biology, Hospital for Sick Children, University of Toronto, Toronto ON, M5G 1X8, Canada

## Abstract

**Background:**

β-catenin and transforming growth factor β signaling are activated in fibroblasts during wound healing. Both signaling pathways positively regulate fibroblast proliferation during this reparative process, and the effect of transforming growth factor β is partially mediated by β-catenin. Other cellular processes, such as cell motility and the induction of extracellular matrix contraction, also play important roles during wound repair. We examined the function of β-catenin and its interaction with transforming growth factor β in cell motility and the induction of collagen lattice contraction.

**Results:**

Floating three dimensional collagen lattices seeded with cells expressing conditional null and stabilized β-catenin alleles, showed a modest negative relationship between β-catenin level and the degree of lattice contraction. Transforming growth factor β had a more dramatic effect, positively regulating lattice contraction. In contrast to the situation in the regulation of cell proliferation, this effect of transforming growth factor β was not mediated by β-catenin. Treating wild-type cells or primary human fibroblasts with dickkopf-1, which inhibits β-catenin, or lithium, which stimulates β-catenin produced similar results. Scratch wound assays and Boyden chamber motility studies using these same cells found that β-catenin positively regulated cell motility, while transforming growth factor β had little effect.

**Conclusion:**

This data demonstrates the complexity of the interaction of various signaling pathways in the regulation of cell behavior during wound repair. Cell motility and the induction of collagen lattice contraction are not always coupled, and are likely regulated by different intracellular mechanisms. There is unlikely to be a single signaling pathway that acts as master regulator of fibroblast behavior in wound repair. β-catenin plays dominant role regulating cell motility, while transforming growth factor β plays a dominant role regulating the induction of collagen lattice contraction.

## Background

Wound healing proceeds through overlapping inflammatory, proliferative and remodeling phases. During the proliferative phase of wound healing, activated fibroblasts induce contraction of the healing wound, move across tissue defects to provide mechanical stability, and act to reorganize the extracellular matrix [1]. These cells persist in hyperplastic wounds and other conditions in which excessive scarring occurs, and as such an understating of their behavior has important practical implications in developing therapies for disorders of wound healing. Although the phenomenon of wound contraction and the reorganization of the extracellular matrix are well recognized, the cellular mechanisms regulating the processes are incompletely understood. These cell processes can be modeled in-vitro by observing the ability of cells to cause contraction of a three-dimensional collagen lattice. Fibroblasts from actively healing wounds have an enhanced ability to cause contraction in these lattices. In the floating collagen lattice contraction assay the ability of fibroblasts to reorganize collagen matrices can be observed[2]. Wound fibroblasts move across the tissue defect to reconstitute the mechanical properties of the damaged tissues[3], a process that can be measured in-vitro using a scratch assay [4-6]. Although several factors are known to regulate fibroblast cell motility, most have been investigated in the context of chemotaxis, using Boyden chambers to measure how they move towards specific agent[7], and there are only a few studies investigating the role of external factors and signaling pathways on fibroblast motility across an injury defect using the scratch assay [5,6].

Transforming growth factor β ligands act through Smad transcription factors to regulate gene expression. These growth factors are expressed during the initial phases of wound repair. Injection of transforming growth factor β – one into cutaneous wounds causes a larger sized wound, and absence of the transforming growth factor β regulated transcription factor, Smad3, results in early wound closure with a smaller wound size [8-10]. Transforming growth factor β – one also enhances the ability of fibroblasts to cause lattice contraction in-vitro [11]. In contrast, its role in modulating fibroblast motility in wound repair is unclear, as although it is demonstrated to act as a chemoattractant for fibroblasts, it has also been shown to inhibit fibroblast motility under other conditions [7,12,13].

Fibroblast-like cells in the dermal compartment of the healing skin during the proliferative phase of wound healing, exhibit high levels of β-catenin protein, and activation of β-catenin mediated tcf dependent transcriptional activity [14,15]. During the normal remodeling phase, β-catenin levels return to baseline, but in human hyperplastic wounds, β-catenin levels remain elevated for a prolonged duration. Studies using genetically modified mice found that β-catenin level correlates with the size of cutaneous wounds. [14,16,17]. β-catenin is a key mediator of the canonical Wnt (wingless) signaling pathway. Canonical Wnt signaling activation results in the stabilization of β-catenin protein. Stabilized cytosolic β-catenin translocates into the nucleus, where it binds to tcf-lef family proteins to form a transcriptional activation complex. Tcf-lef family members are architectural transcription factors, changing DNA conformation when activated. [18-20]. β-catenin also interacts with E-cadherin, and mediates the interplay of adherens junction molecules and the actin cytoskeleton[21]. Stabilization of β-catenin in fibroblast cell cultures increases cell proliferation and invasiveness [15,22,23].

β-catenin has an important role in wound healing. It also mediates the effect of transforming growth factor β in regulating proliferation during wound repair [14,16,17]. As such, it is possible that β-catenin plays a similar role in the regulation of lattice contraction and cell motility during wound repair. We thus examined the role that transforming growth factor β and β-catenin play regulating cell motility and the induction of collagen lattice contraction in primary mouse and human fibroblast cultures.

## Results

### β-catenin negatively regulates the induction of collagen lattice contraction

**Figure 1:**
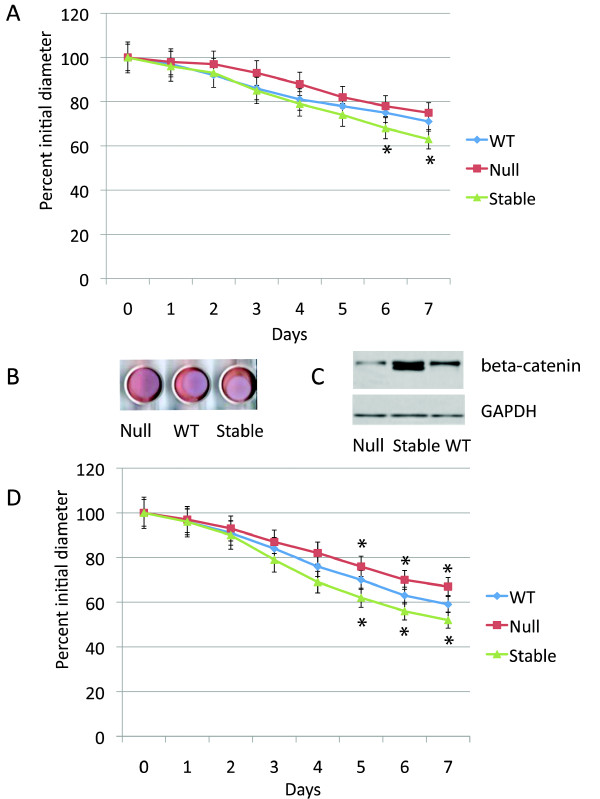
**Collagen lattice contraction is negatively regulated by β-catenin**. A. Means and 95% confidence intervals for collagen lattice mean diameter as observed over seven days are given for fibroblasts from mice expressing the wild type, null, or stabilized β-catenin alleles, all treated with the adenovirus expressing cre recombinase in the absence of serum. There is a statistically significant difference between the stabilized and wild type cells for the time points with an asterisk below the data points. B. Representative photograph of the collagen lattices at day five. C. Western analysis for β-catenin showing successful recombination in the fibroblasts in the lattices. D. Means and 95% confidence intervals for collagen lattice mean diameter as observed over seven days are given for fibroblasts from mice expressing the wild type, null, or stabilized β-catenin alleles, all treated with the adenovirus expressing cre recombinase in the presence of serum. There is a statistically significant difference between either the stabilized or null cells and wild type cells at the time points with an asterisk either above or below the data points.

Primary dermal fibroblast cell cultures were established from mice expressing conditional null or stabilized alleles of β-catenin, and wild type littermates. To activate the conditional alleles, cells were treated with an adenovirus engineered to expresses cre-recombinase[16]. Cells from wild-type mice were also treated with the same adenovirus. Western analysis confirmed the expected difference in β-catenin levels between the samples, with two bands identified for the stabilized form of β-catenin, representing the slightly smaller protein size generated by the conditional allele lacking exon three (Fig. 1C) [24]. Using relative densitometry (to the control protein GAPDH), there was an observed decrease in β-catenin in cells in which the null allele was activated to 32% of control values (p < 0.005), and an increase in β-catenin in levels in cells expressing the stabilized allele to 230% of control value (P < 0.001). There was an observed progressive contraction of the floating collagen lattices over a seven-day period. There was no difference in the rate of lattice contraction between primary dermal fibroblasts derived from wild type mice and those from mice expressing the conditional alleles, but whose alleles had not activated. In cells expressing a stabilized form of β-catenin there was a slightly decreased rate of lattice contraction, while in cells expressing a null form of β-catenin, there was a slightly increased contraction rate when examined in serum free media (Fig. 1A and 1B). The same experiment was performed using media containing serum, and a greater rate of lattice contraction was seen. There was a slightly greater difference in the rate of induction of collagen lattice contraction observed between cells from mice expressing null and stabilized alleles than in experiments undertaken in serum free media (Fig. 1D). To ensure that changes in β-catenin levels did not alter cell numbers, which might change the rate of lattice contraction, we examined the number of cells present using DNA content, and found no difference in cell numbers between the various lattices.

### Transforming growth factor β induced lattice contraction at a substantially greater rate than deficiency in β-catenin

**Figure 2:**
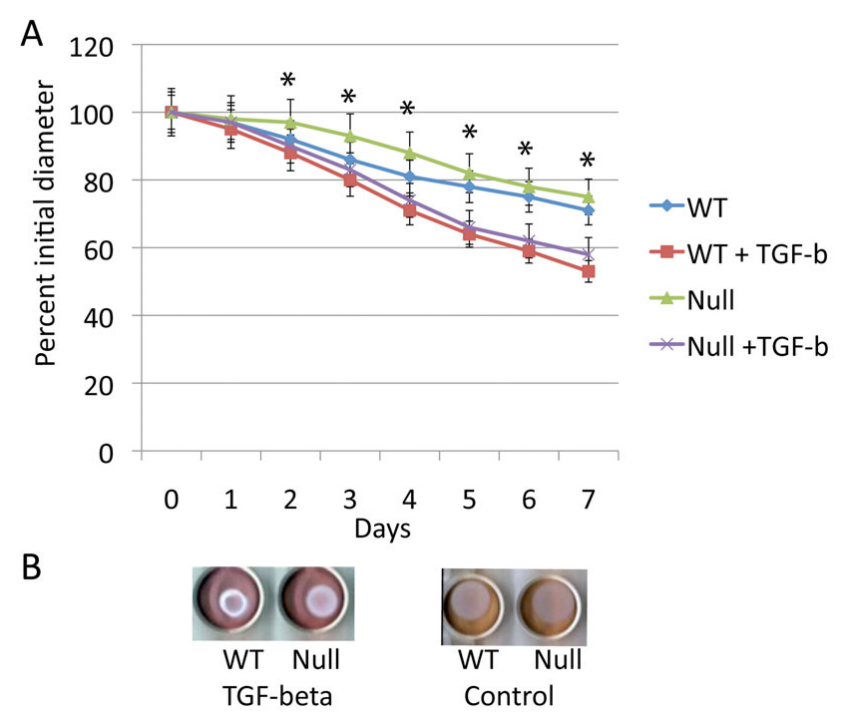
**Transforming growth factor β induces collagen lattice contraction independent of β-catenin**. A Means and 95% confidence intervals for collagen lattice mean diameter as observed over seven days for fibroblasts from mice expressing null β-catenin alleles or wild type littermates treated with the adenovirus expressing cre recombinase also treated with transforming growth factor β, or the carrier. There is a statistically significant difference between transforming growth factor β and carrier treatment for time points identified with an asterisk above the point, but no statistically significant difference between fibroblasts in which the β-catenin null allele was activated compared to treatment with transforming growth factor. Data is shown in the absence of serum. B. Representative photographs of the collagen lattices at day seven.

**Figure 3:**
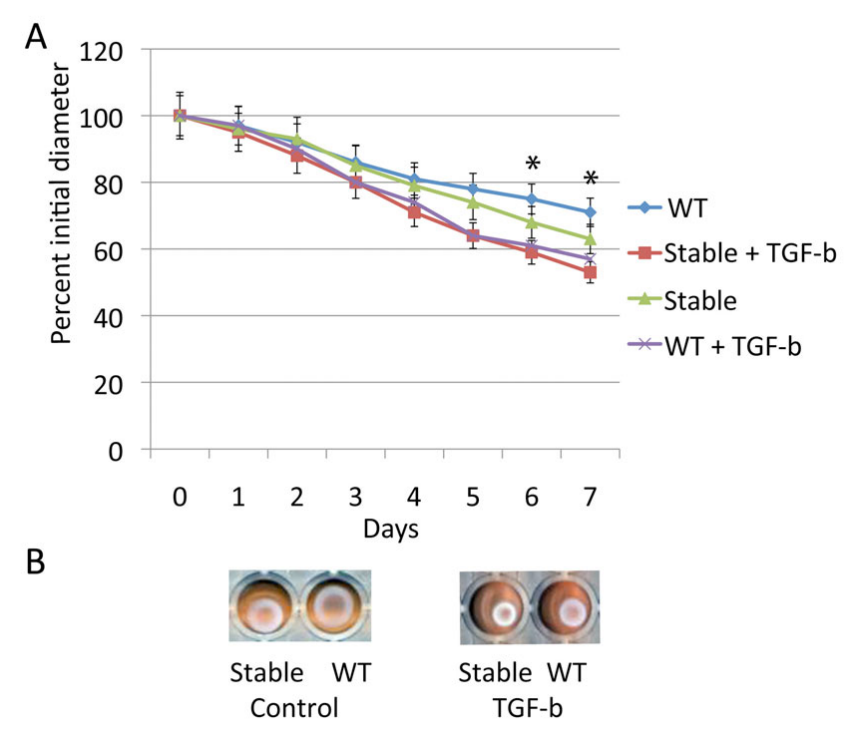
**β-catenin stabilization has a minor effect on collagen lattice contraction compared to transforming growth factor β**. A. Means and 95% confidence intervals for collagen lattice areas as observed over seven days for fibroblasts from mice expressing stabilized β-catenin alleles or wild type littermates treated with the adenovirus expressing cre recombinase, also treated with either transforming growth factor β, or carrier. There is a statistically significant difference between transforming growth factor β and carrier treatment for all time points after day three, and a statistically significant difference between fibroblasts in which the stabilized β-catenin allele was activated compared to fibroblasts from wild type mice for the time points with an asterisk above the data points. Data obtained using serum free media is shown. B. Representative photographs of the collagen lattices at day seven.

Transforming growth factor β one is known to induce contraction of three dimensional collagen lattices seeded with fibroblasts [11]. Since β-catenin partially mediates transforming growth factor β regulated fibroblast cell proliferation[16], we examined if transforming growth factor β induced collagen lattice contraction might be mediated by β-catenin. Contraction rate was compared between wild type and β-catenin null cells treated with transforming growth factor β. The effect of transforming growth factor β induced lattice contraction was not dependent β-catenin (Fig. 2). The relationship of β-catenin stabilization and transforming growth factor β treatment was examined in cells expressing stabilized β-catenin alleles. There was a small effect of β-catenin stabilization compared to transforming growth factor β treatment (Fig. 3). In contrast to its role in fibroblast proliferation, transforming growth factor β induces contraction of collagen lattices independent of β-catenin.

### Lithium and DKK-1 treatment produce similar effects as expression of conditional β-catenin null or stabilized alleles

**Figure 4:**
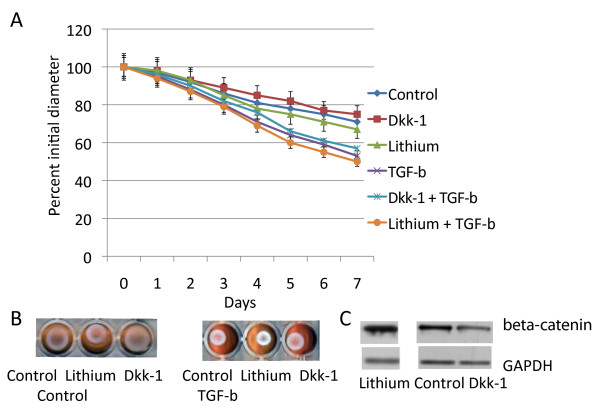
**Dkk-1 and lithium have a minimal effect on collagen lattice contraction**. A. Means and 95% confidence intervals for collagen lattice average diameters as observed over seven days are given for fibroblasts from mice expressing the wild type fibroblasts treated with either an adenovirus expressing Dkk-1 or a control adenovirus. Cultures were also treated with either transforming growth factor β or a carrier. There is a statistically significant difference for transforming growth factor β treatment compared to carrier after day three. For Dkk-1 and lithium treatment there is a minimal change in lattice contraction rate. B. Representative photographs of the collagen lattices at day seven. C. Western analysis for β-catenin showing how Dkk-1 and lithium regulates the protein level of β-catenin.

Lithium will elevate β-catenin level through its regulation of GSK3β [25,26], and dickkopf-1 (Dkk-1) ligand will inhibit Wnt receptor binding, preventing the activation of β-catenin catenin mediated signaling by receptor activation [27,28]. We treated fibroblasts with an adenovirus expressing Dkk-1 [29] and observed their ability of TGF-β to induce lattice contraction. Cell cultures infected with Ad-Dkk-1 demonstrated the same behavior as fibroblasts expressing conditional null alleles of β-catenin (Fig. 4). To determine if lithium could induce fibroblasts to cause lattice contraction, we treated wild type and β-catenin null cells with lithium. Lithium treatment induced β-catenin protein, and inhibited lattice contraction in wild type cell, in a similar manner to that observed in cells expressing β-catenin stabilized conditional alleles (Fig. 4). Lithium and Dkk-1 treatment had no effect on cells expressing null alleles of β-catenin. Using densitometry there was an increase to 195% of baseline β-catenin protein level with lithium treatment (p < 0.01) and a decrease to 45% of control levels with Dkk-1 treatment (P < 0.005).

### Human fibroblasts behave the same as murine cells

**Figure 5:**
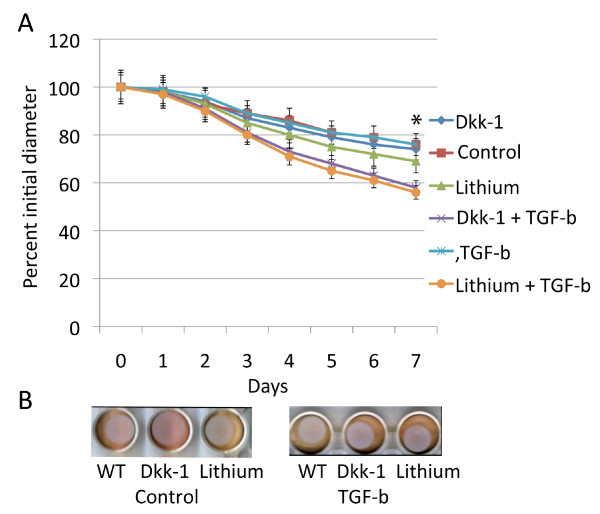
**Human fibroblasts induce collagen lattice contraction in a similar manner as murine firoblasts**. A. Means and 95% confidence intervals for collagen lattice areas as observed over seven days are given for primary cultures from human fibroblasts treated with lithium, Dkk-1, TGF-β, or a carrier. There is a statistically significant difference for TGF-β treatment compared to carrier after day three. For lithium treatment there is a statistically significant difference for the time points with an asterisk above the data points. B. Representative photographs of the collagen lattices at day five.

To determine if human cells behaved the same as cells from mice, we examined human primary fibroblasts in a similar manner. Contraction was compared between cells treated with transforming growth factor β, Dkk-1, lithium, these agents in combination, or with controls. A similar pattern as found in the mouse cultures was observed. Lithium and Dkk-1 have a mild effect on lattice contraction, while transforming growth factor β has a more dramatic positive effect (Fig. 5). Dkk-1 and lithium had similar effects as in murine cultures, showing a mild negative effect of β-catenin on lattice contraction.

### β-catenin, but not transforming growth factor β, positively regulates fibroblast cell motility

**Figure 6:**
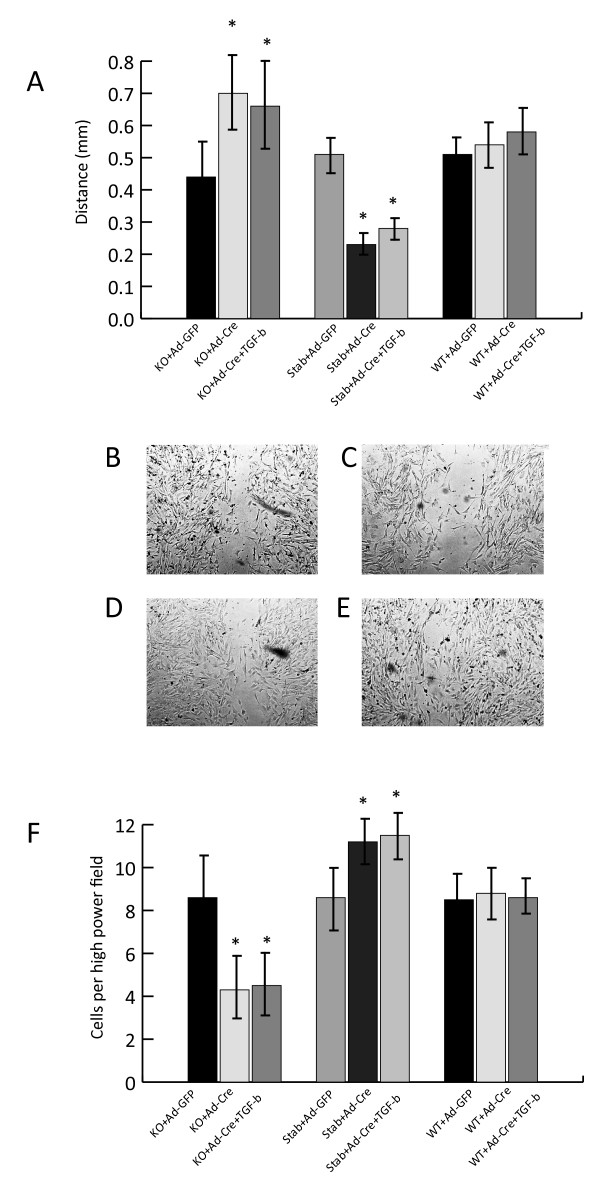
**β-catenin positively regulates cell motility in the scratch assay, while transforming growth factor β has little effect**. A. The means and 95% confidence intervals of the average distance between cells on either side of the scratch (mm). Statistically significant differences (p < 0.05) compared to the controls are indicated with an asterisk above the bar. B through E. Representative photomicrographs of the gap in the cell cultures. B is from wild type cells, C from cells expressing null β-catenin alleles, D from cells treated with transforming growth factor β, and E is from cells expressing stabilized β-catenin alleles. F. The means and 95% confidence intervals of the number of cells passing through the membrane in the Boyden chamber. Statistically significant differences (p < 0.05) compared to the controls are indicated with an asterisk above the bar.

The scratch wound assay can be used to study cell migration, and approximates some of the conditions present during wound repair [4]. Using this assay, we found a positive correlation between β-catenin levels and the rate of cell migration across the scratch wound. Transforming growth factor β had little effect on fibroblast motility using this assay (Fig. 6). Motility was also measured using Boyden chambers. The number of cells moving across the membrane per high powered field correlated with β-catenin level, with cells expressing the stabilized form of β-catenin having an average of 11.2 cell per high powered field, wild type cells 8.6 cells per high powered field, and 4.3 cells per high powered field in cells expressing a null allele of β-catenin (p < 0.01). Transforming growth factor β did not change the number of cells crossing the membrane in the Boyden chamber. In contrast to their ability to induce lattice contraction, β-catenin positively regulates cell motility, while transforming growth factor β plays little role in this process.

### Transforming growth factor β, but not β-catenin, regulates α-smooth muscle actin expression

**Figure 7:**
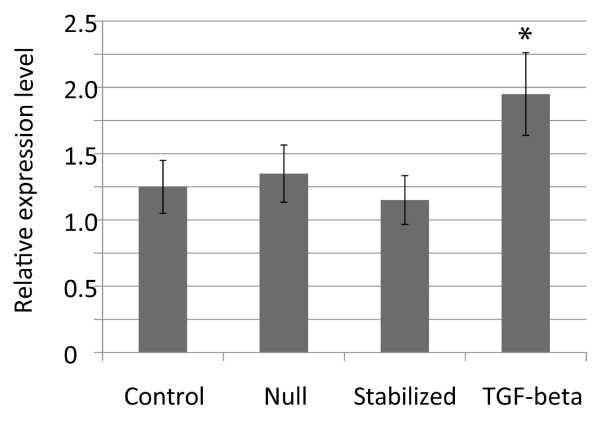
**Transforming growth factor β, but not β-catenin regulates the expression of alpha smooth muscle actin**. Mean and 95% confidence intervals for the relative expression of alpha smooth muscle actin. Treatment with transforming growth factor β resulted in a significantly different level of expression, while activation of conditional alleles of β-catenin had little effect. An asterix above the data point indicates a significant difference from the control value.

α-smooth muscle actin can regulate fibroblast contraction, and the expression of this gene is known to be regulated by transforming growth factor β [30,31]. As such, we examined the regulation of α-smooth muscle actin expression by β-catenin and transforming growth factor β using quantitative RT-PCR in cells grown on plastic tissue culture dishes. Transforming growth factor β treatment increased α-smooth muscle actin expression more than two-fold (Fig. 7). In contrast, the level of expression did not change significantly in cells expressing stabilized or null alleles of β-catenin.

## Discussion

During wound repair, fibroblasts move across tissue defects, and cause contraction of the extracellular matrix. Fibroblast motility is initiated when a cell extends a protrusion at its front which attaches to the substratum on which the cell is migrating, followed by a contraction that moves the cell body forward toward the protrusion, and finally the attachments at the cell rear release as the cell moves forward. The ability of fibroblasts to induce contraction of a collagen lattice is related to activation of fibroblast contractile machinery, the ability to transmit the contractile force to the extracellular matrix, and to the remodeling of the matrix. We found that different signaling pathways regulate these processes, suggesting that these two processes are controlled by different intracellular mechanisms.

Fibroblast activation to myofibroblasts is mediated by α-smooth muscle actin, and can be induced by transforming growth factor β. This is one mechanism by which transforming growth factor β can activate the fibroblast contractile machinery [11,32]. We found that in contrast to transforming growth factor β, β-catenin does not regulate α-smooth muscle actin expression. This finding that is consistent with data from human wound healing. Although α-smooth muscle actin is elevated during the wound healing process, its expression does not vary significantly during the first few weeks of wound repair, a time during which β-catenin level shows substantial variation[14]. Taken together, this suggests that β-catenin mediated tcf-dependent transcription does not directly regulate α-smooth muscle actin expression in fibrobasts.

We found little effect of transforming growth factor β on fibroblast motility. This is in agreement with studies suggesting an inhibitory effect of transforming growth factor β on fibroblast motility [13,33]. However, transforming growth factor β has also been shown to activate cell motility on certain cell surfaces [34]. This suggests that it has different effects depending on the environment in which the fibroblast resides. In contrast to transforming growth factor β, β-catenin was found to positively regulate cell motility. Our findings are in agreement with developmental data, in which canonical Wnt signaling can regulate cell migration, such as in cardiac progenitors, whose migration is controlled by β-catenin signaling [35]. Cell motility is an integral process in wound repair, as cells need to migrate to cross the tissue defect. This is a complex process, during which a cell extends a protrusion at its front, which in turn attaches to the substratum on which the cell is migrating. This is followed by a contraction that moves the cell body forward toward the protrusion, and finally the attachments at the cell rear release as the cell continues to move forward. Chemotactic agents initiate this cycle, and intracellular processes, such as actomyosin filament contraction, which proposes the cell forward, and the formation of adhesive connections in the front, and release of adhesion in the rear of the cell are responsible for propelling the cell. β-catenin participates in adherens junctions, actin cytoskeleton binding, and transcriptional regulation. Participation in each of these processes could regulate cell migration[34].

During wound repair, numerous factors contribute to wound size including the number of cells present (a function of cell proliferation and migration) and the behavior of the cells within the extracellular matrix. Activation of number of signaling pathways, such as through transforming growth factor β and β-catenin, cause a larger wound size. Our data, in concert with data from previous studies, suggests that these two signaling pathways activate different cellular processes to produce a larger wound size. Both transforming growth factor β and β-catenin positively regulate fibroblast proliferation, suggesting that this is a common cellular process in the generation of a hypertrophic wound. In contrast β-catenin has a dominant role regulates cell motility while transforming growth factor β has a dominant role regulating lattice contraction. Such data likely has important implications in therapeutic approaches to hyperplasic wound healing, as the modulation of a multiple involved signaling pathways may be required.

## Conclusion

Cutaneous wound healing is a complex process involving multiple cell types and intracellular signaling pathways. β-catenin and transforming growth factor β play important roles in this process, both of which positively regulate wound size. Here we show that transforming growth factor β plays a major regulatory role, while β-catenin plays a minor role regulating contraction of a floating collagen lattice. In contrast, we found little effect of transforming growth factor β on fibroblast motility, while β-catenin plays a significant role positively regulating fibroblast cell migration. Although β-catenin partially mediates the effect of transforming growth factor β on cell proliferation [16] in fibroblasts, it does not mediate the effect of transforming growth factor β on the induction of contraction of collagen lattices. This demonstrates the complexity of the interaction of various signaling pathways in the regulation of cell behavior in wound repair, that cell motility and the induction of collagen lattice contraction are likely controlled by different intracellular mechanisms, and suggests that there is unlikely to be a single signaling pathway which will act as master regulator of fibroblast behavior in wound repair.

## Methods

### Primary Cell Cultures

Primary cell dermal fibroblast cell cultures were established from mice or from patients undergoing surgery as previously reported[22,36,37]. In the case of human samples, cultures from three independent patients were investigated. All primary cultures were studied within their first three passages. To examine the role of β-catenin, primary fibroblast cell cultures derived from mice expressing β-catenin conditional stabilized or null alleles were utilized. *Catnb*^*tm*2*Kem *^mice contain β-catenin alleles with loxP sites flanking exons 1 and 6. When the segment between loxP sites is excised by exposure to cre-recombinase this effectively abolishes the ability to express β-catenin protein[38]. The *Catnb*^*lox*(*ex*3) ^mouse harbors a conditional β-catenin allele containing loxP sites flanking exon three. When exposed to cre-recombinase, this results in expression of a functional β-catenin protein that is missing the amino terminal phosphorylation sites and as such is a constitutively stabilized, transcriptionally active protein[24]. This research was performed with the approval of an appropriate human and animal ethics committee at our institution.

### Treatments to modulate transforming growth factor β and β-Catenin activity

To drive cre-recombinase expression in the murine fibroblast cells, we used an adenovirus engineered to expresses cre-recombinase (Ad-cre)[16]. Primary cell cultures were infected with 10^8 ^PFU of the virus as in our previous work. In cells from the *Catnb*^*tm*2*Kem *^mouse, this did not completely abolish β-catenin protein level, but reduced the level of to about one third of control levels, and in cells from the *Catnb*^*lox*(*ex*3) ^mouse, treatment doubled the β-catenin protein level. To determine if Wnt ligands are required play a role in the regulation of the lattice contraction, we treated cells with a dickkopf-1 (Dkk1) expressing adenovirus (Ad-Dkk1) as previously reported [29]. Dkk-1 is a potent secreted Wnt antagonist that interacts with Wnt coreceptors of the LRP family[39]. Ad-Dkk1 treatment effectively blocks Wnt mediated signaling during mesenchymal repair processes[40,41]. An adenovirus expressing an empty vector was used as a control. To pharmacologically increase β-catenin protein level, we treated the cells with lithium as previously reported [16,40]. Lithium elevates β-catenin level through regulation of GSK3β[25,26]. Sodium was used as a control. Transforming growth factor β one (Sigma), was added to cultures at a concentration of 10 ng/ml, a concentration that induces maximal effects on the regulation of cell contraction and proliferation in fibroblast cells [42,43].

### Collagen lattice contraction assays

Collagen lattice contraction assays were carried out using murine or human primary cell cultures. The cultures were grown as three dimensional Fibroblast Populated Collagen Lattices (FPLCs). Collagen lattices were prepared by mixing cells with a neutralized solution of collagen type I (8 parts PureCol collagen type I, 2.9 mg/ml, Inamed BioMaterials, Fermont, CA, plus one part 10× α-MEM + 1 part 0.2 M HEPES buffer, pH 9). Final collagen and cell concentrations for the FPCL were 2.0 mg/ml and 3 × 10^5^cells/ml of matrix, respectively. The cell-collagen mixture was aliquoted into 24 well culture dishes (0.5 ml/well) that were pre-treated with a PBS + 2% BSA solution. α-MEM, with or without 10% fetal calf serum (FCS) was added atop FPCLs in each well after polymerization. The attached FPCL were mechanically released from the sides of the culture plates. Digital images of the contracting FPCL were captured at various time points over 7 days using a conventional flatbed Cannon scanner. Average collagen lattice diameter was then measured using imaging software (technique modified from Howard et al [44,45]). At the end of the contraction experiment, the collagen lattices were digested with 1000 units/ml Collagenase I (Worthington -Biochemical Corporation), and cells were isolated and lysed. Total β-Catenin level was examined using western blot, and relative cell number determined using DNA content as previously described [15]. Each individual experiment was performed in at least triplicate, and for each set of conditions the experiment was performed five times. Means, standard deviation, and 95% confidence intervals for the area of the lattices were calculated for each cell type and treatment group, which were then compared using the student t-test.

### Scratch and chemotaxis assays

1.0 × 10^4 ^cells were seeded into 35-mm plastic tissue culture plates. Confluent monolayers were obtained after three days, afterwhich the cells were incubated for 12 hours in serum free media. A "scratch" in the middle of the cell monolayer was produced using a 1 mm wide cell scraper. Cells were observed immediately after the generation of the scratch to ensure a uniform 1 mm wide "scratch" region. They were then observed again 24 hours later to measure the average distance between the cells on each side of the scratch as previously reported [4]. Chemotaxis was measured using primary cell cultures in a modified Boyden chamber as previously reported[36]. A 6-mm Nucleopore membrane (Millipore, Bedford, MA) was placed between the microchemotactic chambers. Cell culture medium with transforming growth factor β, lithium, or Dkk-1 was placed in the lower portion of the chamber. An equal number of cells were placed into the upper chamber for each experiment. Cell migration was determined by counting the number of cells that migrated to the lower portion of the Nucleopore filter over 10 high-power fields. At the end of each experiment the number of cells on the slide (in the case of the scratch assay) or on the top of the Nucleopore membrane in the microchemotactic chambers was counted over ten high powered fields, and no differences in cell numbers were observed between any of the experimental conditions.

### Real time PCR

Real time PCR was used to determine differences in alpha smooth muscle actin using previously reported techniques[46]. Cells derived from genetically modified mice or wild type littermates were examined 24 hours after treatment with the adenovirus or transforming growth factor β. Cells were grown on tissue culture plastic in serum free media for 24 hours. Primers and probes for mouse alpha smooth muscle actin, and 18s rRNA were obtained from Applied Biosystems and used according to the manufacturer's instructions. Quantitative values of alpha smooth muscle actin is normalized based on 18s rRNA content.

## Authors' contributions

RP carried out the collagen contraction assays, SAN, JA, and LS carried out the scrtach and motility assays. BAA concieved conceived of the study, and participated in its design and coordination and drafted the manuscript. All authors read and approved the final manuscript.

## References

[B1] Tomasek JJ, Gabbiani G, Hinz B, Chaponnier C, Brown RA (2002). Myofibroblasts and mechano-regulation of connective tissue remodelling. Nat Rev Mol Cell Biol.

[B2] Shin D, Minn KW (2004). The effect of myofibroblast on contracture of hypertrophic scar. Plast Reconstr Surg.

[B3] Dale PD, Sherratt JA, Maini PK (1997). Role of fibroblast migration in collagen fiber formation during fetal and adult dermal wound healing. Bull Math Biol.

[B4] Liang CC, Park AY, Guan JL (2007). In vitro scratch assay: a convenient and inexpensive method for analysis of cell migration in vitro. Nat Protoc.

[B5] Oberringer M, Meins C, Bubel M, Pohlemann T (2008). In vitro wounding: effects of hypoxia and transforming growth factor beta(1) on proliferation, migration and myofibroblastic differentiation in an endothelial cell-fibroblast co-culture model. J Mol Histol.

[B6] Kopecki Z, Luchetti MM, Adams DH, Strudwick X, Mantamadiotis T, Stoppacciaro A, Gabrielli A, Ramsay RG, Cowin AJ (2007). Collagen loss and impaired wound healing is associated with c-Myb deficiency. J Pathol.

[B7] Postlethwaite AE, Keski-Oja J, Moses HL, Kang AH (1987). Stimulation of the chemotactic migration of human fibroblasts by transforming growth factor beta. J Exp Med.

[B8] Ashcroft GS, Yang X, Glick AB, Weinstein M, Letterio JL, Mizel DE, Anzano M, Greenwell-Wild T, Wahl SM, Deng C (1999). Mice lacking Smad3 show accelerated wound healing and an impaired local inflammatory response. Nat Cell Biol.

[B9] Pierce GF, Mustoe TA, Lingelbach J, Masakowski VR, Griffin GL, Senior RM, Deuel TF (1989). Platelet-derived growth factor and transforming growth factor-beta enhance tissue repair activities by unique mechanisms. J Cell Biol.

[B10] Schreier T, Degen E, Baschong W (1993). Fibroblast migration and proliferation during in vitro wound healing. A quantitative comparison between various growth factors and a low molecular weight blood dialysate used in the clinic to normalize impaired wound healing. Res Exp Med (Berl).

[B11] Montesano R, Orci L (1988). Transforming growth factor beta stimulates collagen-matrix contraction by fibroblasts: implications for wound healing. Proc Natl Acad Sci USA.

[B12] Osornio-Vargas AR, Kalter VG, Badgett A, Hernandez-Rodriguez N, Aguilar-Delfin I, Brody AR (1993). Early-passage rat lung fibroblasts do not migrate in vitro to transforming growth factor-beta. Am J Respir Cell Mol Biol.

[B13] Ellis I, Grey AM, Schor AM, Schor SL (1992). Antagonistic effects of TGF-beta 1 and MSF on fibroblast migration and hyaluronic acid synthesis. Possible implications for dermal wound healing. J Cell Sci.

[B14] Cheon S, Poon R, Yu C, Khoury M, Shenker R, Fish J, Alman BA (2005). Prolonged beta-catenin stabilization and tcf-dependent transcriptional activation in hyperplastic cutaneous wounds. Lab Invest.

[B15] Cheon SS, Cheah AY, Turley S, Nadesan P, Poon R, Clevers H, Alman BA (2002). beta-Catenin stabilization dysregulates mesenchymal cell proliferation, motility, and invasiveness and causes aggressive fibromatosis and hyperplastic cutaneous wounds. Proc Natl Acad Sci USA.

[B16] Cheon SS, Wei Q, Gurung A, Youn A, Bright T, Poon R, Whetstone H, Guha A, Alman BA (2006). Beta-catenin regulates wound size and mediates the effect of TGF-beta in cutaneous healing. Faseb J.

[B17] Sato M (2006). Upregulation of the Wnt/beta-catenin pathway induced by transforming growth factor-beta in hypertrophic scars and keloids. Acta Derm Venereol.

[B18] Clevers H (2006). Wnt/beta-catenin signaling in development and disease. Cell.

[B19] Gordon MD, Nusse R (2006). Wnt signaling: multiple pathways, multiple receptors, and multiple transcription factors. J Biol Chem.

[B20] Moon RT, Kohn AD, De Ferrari GV, Kaykas A (2004). WNT and beta-catenin signalling: diseases and therapies. Nat Rev Genet.

[B21] Brembeck FH, Rosario M, Birchmeier W (2006). Balancing cell adhesion and Wnt signaling, the key role of beta-catenin. Curr Opin Genet Dev.

[B22] Li C, Bapat B, Alman BA (1998). Adenomatous polyposis coli gene mutation alters proliferation through its beta-catenin-regulatory function in aggressive fibromatosis (desmoid tumor). Am J Pathol.

[B23] Soler C, Grangeasse C, Baggetto LG, Damour O (1999). Dermal fibroblast proliferation is improved by beta-catenin overexpression and inhibited by E-cadherin expression. FEBS Lett.

[B24] Harada N, Tamai Y, Ishikawa T, Sauer B, Takaku K, Oshima M, Taketo MM (1999). Intestinal polyposis in mice with a dominant stable mutation of the beta-catenin gene. Embo J.

[B25] Hedgepeth CM, Conrad LJ, Zhang J, Huang HC, Lee VM, Klein PS (1997). Activation of the Wnt signaling pathway: a molecular mechanism for lithium action. Dev Biol.

[B26] Stambolic V, Ruel L, Woodgett JR (1996). Lithium inhibits glycogen synthase kinase-3 activity and mimics wingless signalling in intact cells. Curr Biol.

[B27] Bafico A, Liu G, Yaniv A, Gazit A, Aaronson SA (2001). Novel mechanism of Wnt signalling inhibition mediated by Dickkopf-1 interaction with LRP6/Arrow. Nat Cell Biol.

[B28] Fedi P, Bafico A, Nieto Soria A, Burgess WH, Miki T, Bottaro DP, Kraus MH, Aaronson SA (1999). Isolation and biochemical characterization of the human Dkk-1 homologue, a novel inhibitor of mammalian Wnt signaling. J Biol Chem.

[B29] Kuhnert F, Davis CR, Wang HT, Chu P, Lee M, Yuan J, Nusse R, Kuo CJ (2004). Essential requirement for Wnt signaling in proliferation of adult small intestine and colon revealed by adenoviral expression of Dickkopf-1. Proc Natl Acad Sci USA.

[B30] Hinz B, Celetta G, Tomasek JJ, Gabbiani G, Chaponnier C (2001). Alpha-smooth muscle actin expression upregulates fibroblast contractile activity. Mol Biol Cell.

[B31] Vaughan MB, Howard EW, Tomasek JJ (2000). Transforming growth factor-beta1 promotes the morphological and functional differentiation of the myofibroblast. Exp Cell Res.

[B32] Hinz B (2007). Formation and function of the myofibroblast during tissue repair. J Invest Dermatol.

[B33] Mii S, Ware JA, Kent KC (1993). Transforming growth factor-beta inhibits human vascular smooth muscle cell growth and migration. Surgery.

[B34] Thampatty BP, Wang JH (2007). A new approach to study fibroblast migration. Cell Motil Cytoskeleton.

[B35] Yue Q, Wagstaff L, Yang X, Weijer C, Munsterberg A (2008). Wnt3a-mediated chemorepulsion controls movement patterns of cardiac progenitors and requires RhoA function. Development.

[B36] Li C, Nguyen Q, Cole WG, Alman BA (2001). Potential treatment for clubfeet based on growth factor blockade. J Pediatr Orthop.

[B37] Tejpar S, Li C, Yu C, Poon R, Denys H, Sciot R, Van Cutsem E, Cassiman JJ, Alman BA (2001). Tcf-3 expression and beta-catenin mediated transcriptional activation in aggressive fibromatosis (desmoid tumour). Br J Cancer.

[B38] Brault V, Moore R, Kutsch S, Ishibashi M, Rowitch DH, McMahon AP, Sommer L, Boussadia O, Kemler R (2001). Inactivation of the beta-catenin gene by Wnt1-Cre-mediated deletion results in dramatic brain malformation and failure of craniofacial development. Development.

[B39] Niehrs C (2006). Function and biological roles of the Dickkopf family of Wnt modulators. Oncogene.

[B40] Chen Y, Whetstone HC, Lin AC, Nadesan P, Wei Q, Poon R, Alman BA (2007). Beta-catenin signaling plays a disparate role in different phases of fracture repair: implications for therapy to improve bone healing. PLoS Med.

[B41] Chen Y, Whetstone HC, Youn A, Nadesan P, Chow EC, Lin AC, Alman BA (2007). Beta-catenin signaling pathway is crucial for bone morphogenetic protein 2 to induce new bone formation. J Biol Chem.

[B42] Brown RA, Sethi KK, Gwanmesia I, Raemdonck D, Eastwood M, Mudera V (2002). Enhanced fibroblast contraction of 3D collagen lattices and integrin expression by TGF-beta1 and -beta3: mechanoregulatory growth factors?. Exp Cell Res.

[B43] Yang CC, Lin SD, Yu HS (1997). Effect of growth factors on dermal fibroblast contraction in normal skin and hypertrophic scar. J Dermatol Sci.

[B44] Howard JC, Varallo VM, Ross DC, Roth JH, Faber KJ, Alman B, Gan BS (2003). Elevated levels of beta-catenin and fibronectin in three-dimensional collagen cultures of Dupuytren's disease cells are regulated by tension in vitro. BMC Musculoskelet Disord.

[B45] Tse R, Howard J, Wu Y, Gan BS (2004). Enhanced Dupuytren's disease fibroblast populated collagen lattice contraction is independent of endogenous active TGF-beta2. BMC Musculoskelet Disord.

[B46] Gallucci RM, Lee EG, Tomasek JJ (2006). IL-6 modulates alpha-smooth muscle actin expression in dermal fibroblasts from IL-6-deficient mice. J Invest Dermatol.

